# Post-collection acidification of spot urine sample is not needed before measurement of electrolytes

**DOI:** 10.11613/BM.2022.020702

**Published:** 2022-04-15

**Authors:** Tomáš Šálek, Pavel Musil, Marek Pšenčík, Vladimír Palička

**Affiliations:** 1 Institute of Clinical Biochemistry and Diagnostics, Medical Faculty in Hradec Králové, Charles University, Prague, Czech Republic; 2 Department of Clinical biochemistry and pharmacology, The Tomas Bata Hospital in Zlín, Zlín, Czech Republic

**Keywords:** urolithiasis, calcium, magnesium, phosphorus, preanalytical phase

## Abstract

**Introduction:**

Kidney stone formers can have higher oxalate and phosphate salt amounts in their urine than healthy people and we hypothesized that its acidification may be useful. The study aims to compare results of urine concentrations of calcium, magnesium, and inorganic phosphorus in the midstream portion of first voided morning urine samples without (FMU) and with post-collection acidification (FMUa) in kidney stone patients.

**Materials and methods:**

This is a prospective single center study. A total of 138 kidney stone patients with spot urine samples were included in the study. Urine concentrations of calcium, magnesium and inorganic phosphorus were measured with and without post-collection acidification. Acidification was performed by adding 5 µL of 6 mol/L HCl to 1 mL of urine.

**Results:**

The median age (range) of all participants was 56 (18-87) years. The median paired differences between FMU and FMUa concentrations of calcium, magnesium, and inorganic phosphorus were: - 0.040 mmol/L, 0.035 mmol/L, and 0.060 mmol/L, respectively. They were statistically different: P < 0.001, P < 0.001, P = 0.004, respectively. These differences are not clinically significant because biological variations of these markers are much higher.

**Conclusions:**

No clinically significant differences in urinary calcium, magnesium, and inorganic phosphorus concentrations between FMU and FMUa in patients with kidney stones were found.

## Introduction

The recurrent kidney stone disease is a major clinical and economic problem (*1*). Metaphylaxis (secondary prevention) of urolithiasis is the way of prevention of recurrent kidney stones development (*2*). The medical laboratory plays a key role in the diagnosis and monitoring of kidney stone risk factors. These include increased urine osmolality, sodium, calcium, inorganic phosphorus, and oxalate concentrations. Urinary citrate and magnesium inhibit kidney stones formation, their low concentrations also lead to stone formation. Low urine volume itself is the risk factor for kidney stone formation (*3*).

Urine risk factors can be tested in random morning samples and in 24-hour urine collections. The 24-hour urine collections have higher information value but they are not convenient for patients (*4*). The 24-hour urine sample is more recommended due to diurnal variations in urine pH and biological variation of kidney stone formation risk factor concentrations. Antibacterial preservatives such as thymol or toluene are used for 24-hour collections in kidney stone patients (*5*). Ideally, the samples should be stored at 4 °C. When HCl is used as a preservative, it seems essential to neutralize samples before analysis (*6*). On the other hand, the first morning urine (FMU) is the most concentrated and is recommended for crystalluria detection due to the reduced water intake during night (*7*).

In general, acidification of urine sample is aimed at inhibiting microbial growth. This is particularly relevant for 24-hour urine collection that is not refrigerated. It is less applicable to spot urine that is processed within 1 hour of collection. Acidification serves to prevent the precipitation of calcium oxalate and calcium phosphate salts. Calcium and magnesium tend to precipitate with phosphate mainly within alkaline pH range. However, post-collection preanalytical urine acidification, centrifugation, and heat incubation of urine samples does not have clinically significant effect on calcium, magnesium, and inorganic phosphorus measurement when biological variation is taken into account (*8*). However, the studies were performed on healthy people or the general population (*8*, *9*). We hypothesized that kidney stone formers can have higher oxalate and phosphate salt amounts in their urine than healthy people and acidification may be useful. The need for acidification would be more visible in this special subgroup of kidney stone patients. In routine practice, acidification of urine would be implemented to all urine samples delivered to the laboratory with the requested measurement of calcium, magnesium, and inorganic phosphorus. The aim of the study is to compare results of urine concentrations of calcium, magnesium, and inorganic phosphorus in mid-stream first voided morning urine samples without (FMU) and with post-collection acidification (FMUa) in kidney stone patients.

## Materials and methods

### Subjects

A total of 138 consecutive kidney stone patients from Metabolic Clinic were considered for the participation in a prospective single center study. The only inclusion criterion was kidney stone diagnosis. No patients were excluded from the study. Metabolic Clinic is a part of the Department of Clinical biochemistry and pharmacology, and represents its clinical practice. The Metabolic Clinic focuses on the metaphylaxis of urolithiasis. All included patients were diagnosed with a kidney stone diagnosis based on clinical examination, radiological findings, sonography findings, and kidney stone analysis results at the Urology Clinic. These patients were referred to the Metabolic Clinic for long term metaphylaxis of urolithiasis. Their urinary kidney stones risk factors are monitored on regular basis two times a year. The diagnosis of kidney stones (N200) according to the International Statistical Classification of Diseases and Related Health Problems, the tenth revision (ICD-10) was also included at a request form from Metabolic Clinic in all patients.

### Methods

First morning urine samples were taken in 10 mL polypropylene urine tube without preservatives (FL Medical, Padua, Italy). The post-collection acidification of aliquot sample (FMUa) was performed by adding 5 µL of 6 mol/L HCl to 1 mL of urine (*10*). Urine concentrations of calcium, magnesium, and inorganic phosphorus were measured in all samples by automated methods on the same Abbott Architect analyser ci16200 (Abbott Laboratories, Illinois, USA) within one hour after acceptance of urine by laboratory staff.

All tests were analysed by a single measurement. The laboratory methods are regularly evaluated by internal and external quality control assessment. External quality control is performed by external quality control provider SEKK s.r.o. (www.sekk.cz). Internal quality control material (catalogue number AU 2352) was manufactured by RANDOX (Randox Laboratories Limited, County Antrim, United Kingdom). Internal quality material was measured daily before patient samples measurement. All data are displayed by Levey-Jennings charts due to trend visibility. The quality control result is always rejected when it exceeds three standard deviations from the mean. Many calculated analytical performance characteristics are included in quality management: intermediate precision, bias, total analytical error, sigma metrics *etc.* The sigma metrics of all three methods is better than six.

The mean differences between FMU and FMUa were compared to the intraindividual biological variation (CV_w_) data of corresponding analytes: calcium 27.5%, magnesium 45.4%, inorganic phosphorus 26.4% (*11*). The mean differences higher than biological variations were considered significant.

Samples with urine pH ≥ 6.8 were analysed separately, because we hypothesized that, in pH range above second dissociation constant of phosphoric acid (6.8), even more calcium phosphate salts could be dissolved by acidification with resulting increased urine concentrations of calcium and phosphate (*12*).

### Statistical analysis

The normality of data distribution was assessed by D’Agostino-Pearson test. Data did not have normal distribution. Non-parametric tests were used for further data analysis. Wilcoxon paired samples test was used for comparison of median differences and zero. Bland-Altman plot was used for visualization of differences. Bland-Altman plot includes line of equality, line of mean difference with 95% confidence interval, regression line of differences with 95% confidence interval.

The calculations were performed using MedCalc statistical software version 20.015 (MedCalc Software bvba, Ostend, Belgium). The difference was statistically significant when P < 0.050. The level of significance was written with three decimal places (*13*).

The study was approved by the local hospital Ethics committee No. 2021-69. All patients signed the informed consent with anonymous publication of their data.

## Results

The median age (range) of all participants was 56 (18–87) years. There were 83 males and 55 females.

**Figure 1:**
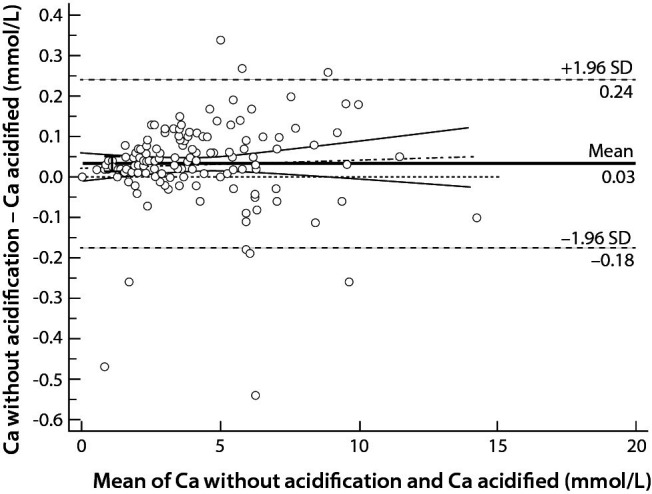
Bland-Altman plot: Comparison between urine calcium (Ca) concentrations in samples without and with acidification in all samples. Dotted line – line of equality. Solid line – line of mean difference. Dash-dotted line - regression line of differences with 95% confidence interval (presented as continuous lines). SD - standard deviation.

**Figure 2:**
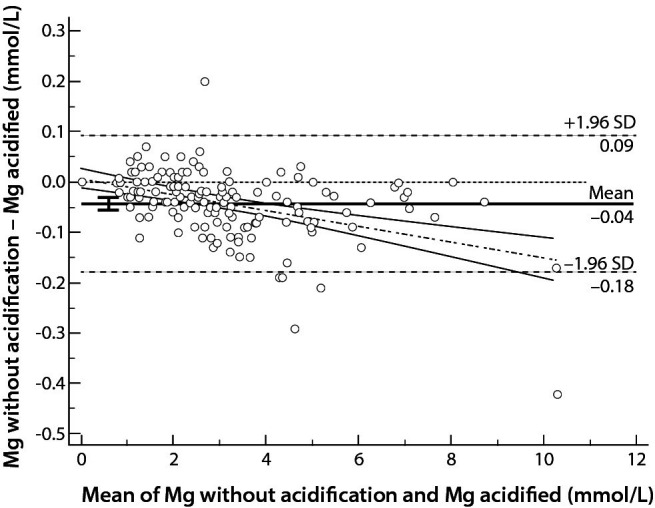
Bland-Altman plot: Comparison between urine magnesium (Mg) concentrations in samples without and with acidification in all samples Dotted line – line of equality. Solid line – line of mean difference. Dash-dotted line - regression line of differences with 95% confidence interval (presented as continuous lines). SD - standard deviation.

**Figure 3:**
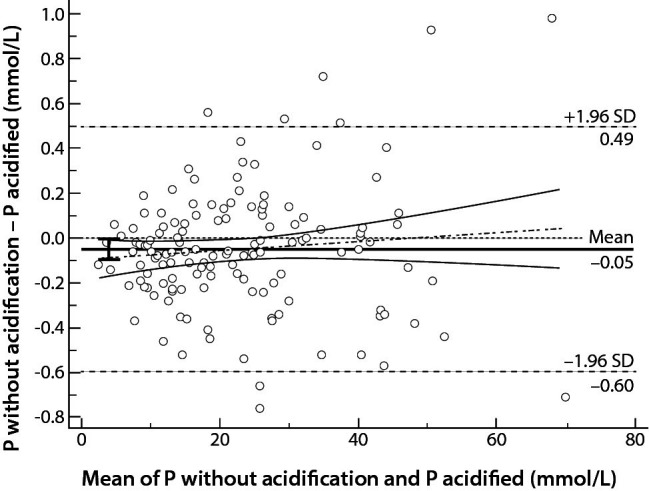
Bland-Altman plot: Comparison between urine inorganic phosphorus (P) concentrations in samples without and with acidification in all samples. Dotted line – line of equality. Solid line – line of mean difference. Dash-dotted line - regression line of differences with 95% confidence interval (presented as continuous lines). SD - standard deviation.

The results of concentrations of urine calcium, magnesium and inorganic phosphorus in acidified and non-acidified samples are shown in Table 1. The differences between acidified and non-acidified samples are showed in Bland-Altman plots (Figures 1-3). The arithmetic means of FMU calcium, FMU magnesium, and FMU inorganic phosphorus were 4.06 mmol/L, 3.14 mmol/L, and 22.78 mmol/L, respectively. The mean paired differences between FMU and FMUa for calcium, magnesium, and inorganic phosphorus were 0.7%, 1.3%, 0.2%, respectively. All mean differences weree lower than the corresponding intraindividual biological variabilities (calcium 27.5%, magnesium 45.4%, inorganic phosphorus 26.4%).

**Table 1:** Results of urine calcium, magnesium, and inorganic phosphorus in samples without and with acidification

	**Without acidification**	**With acidification**	**Hodges-Lehmann ** **median difference**	**P**	**Types of kidney stones (N)**
Calcium, mmol/L (all 138 samples)	3.60 (2.26-5.78)	3.52 (2.23-5.64)	- 0.040	P < 0.001	100% CaOx stones (62) 100% CaP Stones (3) Mixed CaOx and CaP stones (41) Mixed CaOx, CaP and struvite stones (2) Mixed CaP and struvite (1) Mixed CaOx and ammonium urate stones (1) Mixed CaOx and Uric acid stones (2) Uric acid (10) Cystin (1) Unknown composition (15)
Magnesium, mmol/L (all 138 samples)	2.77 (1.84-4.21)	2.81 (1.87-4.24)	0.035	P < 0.001	
Inorganic phosphorus, mmol/L (all 138 samples)	20.81 (12.84-29.55)	20.70 (12.95-29.02)	0.060	P = 0.004	
Calcium, mmol/L (20 samples with pH ≥ 6.8)	2.35 (1.44-3.88)	2.29 (1.52-3.75)	- 0.040	P = 0.007	100% CaOx stones (10) 100% CaP Stones (2) Mixed CaOx and CaP stones (6) Mixed CaP and struvite (1) Uric acid (1) (ongoing infection)
Magnesium, mmol/L (20 samples with pH ≥ 6.8)	2.10 (0.97-3.15)	2.10 (0.95-3.15)	0.000	P = 0.594	
Inorganic phosphorus, mmol/L (20 samples with pH ≥ 6.8)	12.30 (8.92-18.14)	12.47 (9.09-18.57)	0.198	P < 0.001	
Results are presented as median (interquartile range). CaOx - Calcium oxalate. CaP - Calcium phosphate. N - number of samples.					

## Discussion

No clinically significant differences in urinary calcium, magnesium, and inorganic phosphorus concentrations between FMU and FMUa in patients with kidney stones were found in either whole group or in subgroup with urine pH ≥ 6.8.

Feres *et al.* showed that the acidification of urine in the laboratory with addition of 5 mL of 6 mol/L HCl to 1 L of urine led to lower mean concentration of calcium and increased mean concentrations of both magnesium and inorganic phosphorus (*10*). Our results had the same trends and we added the same proportion of 6 mol/L HCl. Larcher *et al.* reported that urine acidification before calcium measurement is necessary in patients with urinary crystals (*14*). Our patients received preventive treatment (metaphylaxis) and probably were without significant number of urine crystals.

Ricos *et al.* reported intraindividual biological variations of urine calcium, magnesium and inorganic phosphorus in 24-hour urine collections (*11*). The EFLM biological variation database is another important source of information on biological variation. The differences, in urine concentrations of calcium, magnesium, and inorganic phosphorus, between acidified and non-acidified samples in our kidney stone patients were much lower than their intraindividual biological variations.

Yilmaz *et al.* showed no effect of urine acidification in the laboratory after 24-hour collection without preservatives for the measurement of calcium, magnesium and phosphate (*15*). This study supports our results.

The consensus statement by Williams *et al.* reported that both 24-hour urine collection and spot urine samples are useful for identification and monitoring of patients with kidney stones. Microscopic evaluation of urinary crystals would be valuable for the assessment of prediction of the recurrent kidney stone disease (*16*). Our patients are monitored by both 24-hour urine collections and spot urine samples. First morning urine samples are obtained more frequently, because 24-hour urine collection is inconvenient and many patients dislike it.

Darn *et al.* reported that at a pH greater than 6.5 measured calcium, magnesium and phosphate significantly decreased as a result of salt precipitation. These changes were not significant clinically (*17*). We also did not find clinically significant changes due to acidification.

The limitation of our work is that we did not examine the urine samples for the presence of crystals because crystals reflect urine supersaturation (*7*). The small sample size is another limitation because higher number of participants could better explain statistical relations. Some rare types of kidney stones were not included in the study.

## Conclusions

No clinically significant differences in urinary calcium, magnesium, and inorganic phosphorus concentrations between FMU and FMUa in patients with kidney stones were found. Acidification of urine was neither useful in the subgroup of patients with urine pH ≥ 6.8.

## Data Availability

The data generated and analysed in the presented study are available from the corresponding author on request.

## References

[1] Ziemba JB Matlaga BR. Epidemiology and economics of nephrolithiasis. Investig Clin Urol. 2017;58:299–306. 10.4111/icu.2017.58.5.299 28868500 PMC5577325

[2] Fisang C Anding R Müller SC Latz S Laube N. Urolithiasis-an Interdisciplinary Diagnostic, Therapeutic and Secondary Preventive Challenge. Dtsch Arztebl Int. 2015;112:83–91. 10.3238/arztebl.2015.0083 25721435 PMC4349965

[3] European Association of Urology (EAU). EAU guidelines on urolithiasis. Available from: https://uroweb.org/guideline/urolithiasis/#4. Accessed: July 2nd 2021.

[4] Strohmaier WL Hoelz KJ Bichler KH. Spot urine samples for the metabolic evaluation of urolithiasis patients. Eur Urol. 1997;32:294–300. 10.1159/000480828 9358216

[5] Šálek T. Extreme diet without calcium may lead to hyperoxaluria and kidney stone recurrence-A case study. J Clin Lab Anal. 2020;34:e23512. 10.1002/jcla.23512 32761639 PMC7755765

[6] Wu W Yang D Tiselius H-G Ou L Mai Z Chen K Collection and storage of urine specimens for measurement of urolithiasis risk factors. Urology. 2015;85:299–303. 10.1016/j.urology.2014.10.030 25623670

[7] Daudon M Frochot V. Crystalluria. Clin Chem Lab Med. 2015;53:s1479–87. 10.1515/cclm-2015-0860 26509782

[8] Sodi R Godber IM. Effect of refrigeration, centrifugation, acidification, heat treatment and storage on urine calcium, magnesium and phosphate. Clin Chem Lab Med. 2016;54:e379–81. 10.1515/cclm-2016-0064 27155007

[9] Chenevier-Gobeaux C Rogier M Dridi-Brahimi I Koumakis E Cormier C Borderie D. Pre-, post- or no acidification of urine samples for calcium analysis: does it matter? Clin Chem Lab Med. 2019;58:33–9. 10.1515/cclm-2019-0606 31539348

[10] Feres MC Bini R De Martino MC Biagini SP de Sousa AL Campana PG Implications for the use of acid preservatives in 24-hour urine for measurements of high demand biochemical analytes in clinical laboratories. Clin Chim Acta. 2011;412:2322–5. 10.1016/j.cca.2011.08.033 21910978

[11] Ricós C Alvarez V Cava F García-Lario JV Hernández A Jiménez CV Current databases on biological variation: pros, cons and progress. Scand J Clin Lab Invest. 1999;59:491–500. 10.1080/00365519950185229 10667686

[12] Ennis JL Asplin JR. The role of the 24-h urine collection in the management of nephrolithiasis. Int J Surg. 2016;36:633–7. 10.1016/j.ijsu.2016.11.020 27840312

[13] Simundic AM. Practical recommendations for statistical analysis and data presentation in Biochemia Medica journal. Biochem Med (Zagreb). 2012;22:15–23. 10.11613/BM.2012.003 22384516 PMC4062332

[14] Larcher L Lefevre G Bailleul S Daudon M Frochot V. Importance of pre-analytical for urinalysis with urinary crystals. Ann Biol Clin (Paris). 2017;75:525–30. 10.1684/abc.2017.1278 28958961

[15] Yilmaz G Yilmaz FM Hakligör A Yücel D. Are preservatives necessary in 24-hour urine measurements? Clin Biochem. 2008;41:899–901. 10.1016/j.clinbiochem.2008.03.002 18371307

[16] Williams JC Gambaro G Rodgers A Asplin J Bonny O Costa-Bauzá A Urine and stone analysis for the investigation of the renal stone former: a consensus conference. Urolithiasis. 2021;49:1–16. 10.1007/s00240-020-01217-3 33048172 PMC7867533

[17] Darn SM Sodi R Ranganath LR Roberts NB Duffield JR. Experimental and computer modelling speciation studies of the effect of pH and phosphate on the precipitation of calcium and magnesium salts in urine. Clin Chem Lab Med. 2006;44:185–91. 10.1515/CCLM.2006.034 16475905

